# Analytical and Experimental Performance Evaluation of BLE Neighbor Discovery Process Including Non-Idealities of Real Chipsets

**DOI:** 10.3390/s17030499

**Published:** 2017-03-03

**Authors:** David Perez-Diaz de Cerio, Ángela Hernández, Jose Luis Valenzuela, Antonio Valdovinos

**Affiliations:** 1 Signal Theory and Communications Department, Universitat Politècnica de Catalunya, C/Esteve Terrades 7, 08860 Castelldefels, Spain; valens@tsc.upc.edu; 2 Aragon Institute for Engineering Research (I3A), University of Zaragoza, 50018 Zaragoza, Spain; anhersol@unizar.es (Á.H.); toni@unizar.es (A.V.)

**Keywords:** Bluetooth Low Energy, neighbor discovery, non-detection probability, discovery latency, real testbed

## Abstract

The purpose of this paper is to evaluate from a real perspective the performance of Bluetooth Low Energy (BLE) as a technology that enables fast and reliable discovery of a large number of users/devices in a short period of time. The BLE standard specifies a wide range of configurable parameter values that determine the discovery process and need to be set according to the particular application requirements. Many previous works have been addressed to investigate the discovery process through analytical and simulation models, according to the ideal specification of the standard. However, measurements show that additional scanning gaps appear in the scanning process, which reduce the discovery capabilities. These gaps have been identified in all of the analyzed devices and respond to both regular patterns and variable events associated with the decoding process. We have demonstrated that these non-idealities, which are not taken into account in other studies, have a severe impact on the discovery process performance. Extensive performance evaluation for a varying number of devices and feasible parameter combinations has been done by comparing simulations and experimental measurements. This work also includes a simple mathematical model that closely matches both the standard implementation and the different chipset peculiarities for any possible parameter value specified in the standard and for any number of simultaneous advertising devices under scanner coverage.

## 1. Introduction

Bluetooth is a technology usually associated with short-range personal area networks with a low density of active devices, and many users only see it as the technology employed by their hands-free devices [1,2]. However, this vision of Bluetooth is changing with the appearance of the Internet of Things (IoT), connecting billions of devices and services anytime and everywhere, generating an incredible amount of new applications in many fields: wearable devices, health care, smart homes, connected cars, smart cities, etc. [3,4].

Some of the IoT applications [5], which require mobility and global connectivity based on a cellular network, will use 4G technologies such as LTE Machine to Machine (LTE-M) or Narrowband IoT (NB-IoT) and 5G technologies in the future. Nevertheless, most of the IoT devices will not use cellular wireless systems; instead, they will employ technologies that operate in Industrial Scientific Medical (ISM) unlicensed bands, such as Wi-Fi, ZigBee, Bluetooth and others, which will form what is known as capillary networks [6]. This is one of the reasons why Bluetooth introduced from its Version 4.0 [7] a low energy variant known as Bluetooth Smart or Bluetooth Low Energy (BLE). BLE includes lower power modes, lower complexity and lower cost, which are indispensable characteristics for making it a suitable technology for the IoT in the following years.

As any emerging technology, ble has raised a wide range of new questions and aspects that need to be characterized: its power efficiency, latency, range, throughput, etc. Searching for a global perspective that takes into account all of them for the whole variety of ble-based services and applications is practically unapproachable. In [8], the ble protocol stack is described, and the impact of several parameters on its performance is investigated, showing that configuration parameters have a big impact on the tradeoff between performance and energy consumption. Many other studies evaluate new services and applications based on the ble technology: healthcare, home automation, proximity sensing, consumer electronics, security, vehicular communications, sport, etc. Some examples are the ble-based blood pressure monitoring system developed in [9], the remote lock system designed in [10] for pervasive computing environments, the fuzzy logic-based mechanism for energy management in home automation presented in [11] or the studies about the potential feasibility of ble technology for intra-vehicular wireless sensor networks [12] and for inter-vehicular communications [13]. Among that range of applications, we focus our analysis on high density scenarios. These involve the discovery of a large number of users/devices in a short period of time, such as race tracking for sport events, cattle control, access control, goods traceability, etc.

The purpose of this paper is to evaluate the performance of ble as a technology that enables fast and reliable discovery and identification in high density environments from a real perspective, including the non-idealities present in off-the-shelf devices. The probability of not discovering a device in a given period of time depends on a vast number of variables and parameters. Some of them are technology dependent, such as the scan window, scan interval, advertising interval, frame size, advertising channels, transmitted power, etc., and others come determined by the environment: interference, noise, collisions, received signal level, etc. In this paper, we will analyze the probability of not detecting a frame of a system with multiple advertisers (devices transmitting beacon frames periodically) and one device continuously scanning for those frames. Once we obtain this probability, several other metrics will be derived, which result in being very useful for the IoT world, such as the average number of transmissions before detection, the detection latency, the average number of detections within a window of opportunity, etc.

The importance of the topic and its expected impact in the following years is revealed by previous works in the literature, which focus on similar aspects, such as the modeling of the discovery latency. For example, [14] proposes an analytical model for evaluating a three-channel-based neighbor discovery procedure, further enhanced in [15] with real experiments to analyze the energy consumption. The work in [8] evaluates the impact of several of its parameters on its performance, including also real measurements of the energy consumption. Another example of the analysis of the latency performance combined with simulation experiments can be found in [16], where multiple ble pairs (advertisers and scanners) are considered.

The most recent work in [17] improves the models of [14,15] using the Chinese remainder theorem. This work analyzes the best tradeoff between discovery latency and energy consumption considering a variable scan window, scan interval and advertising intervals and concluding that the best configuration comes when the advertising interval is equal to the scan window.

All of the aforementioned works are based on the premise that the scanner behavior matches exactly what is derived from the standard and do not consider non-idealities present in real devices. For example, one could consider that when the scan window is matched with the scan interval, the scanner should be doing a continuous scan, i.e., scanning 100% of the time. However, along the paper, we will demonstrate that this is not true, and the impact of these non-idealities is very significant on the results and calculations derived from them. Measurements show that additional gaps appear in the scanning process, which reduce the discovery capabilities. These gaps have been identified in all of the measured devices and respond to both regular patterns and variable events associated with the decoding process. In addition, we propose a simple mathematical model for the non-detection probability and the main performance parameters derived from it, which includes both the standard implementation and the non-idealities present in the devices. The results have been validated with extensive experimentation in a real testbed, and simulations have been carried out to extrapolate the results for high density scenarios. The mathematical model will be a valuable tool to easily obtain an upper bound of discovery capacities and to select the desirable parameters values according to a particular BLE application.

The structure of the paper is as follows. In Section 2, we shortly describe the standard BLE, and we present the experimental analysis performed in order to characterize the non-idealities existing in the manufactured chipset scanner operation. In Section 3, we propose a simple mathematical model that meets both the standard implementation and the manufactured chipset peculiarities. Numerical results from the mathematical model, the simulation and the experimental measurements of real devices are presented and discussed in Section 4. Finally, the main conclusions are summarized in Section 5.

## 2. BLE Overview

BLE was first introduced in Version 4.0 of the Bluetooth Core Specification [7]. The low energy system includes features designed to enable products that require lower current consumption, less complexity and reduced cost compared to Bluetooth legacy products. The system is also designed for use cases and applications with lower data rates and has shorter duty cycles. Like legacy Bluetooth, the BLE radio operates in the unlicensed 2.4 GHz ISM band using forty physical channels separated by 2 MHz. The neighbor discovery process is one of the main modifications that were introduced in BLE. Unlike classical Bluetooth, BLE dedicates three special channels (37, 38 and 39), called advertising channels, to the discovery of other BLE devices, initiating a connection and broadcasting data. Channels from 0 to 36 are used for data exchange between connected devices. The BLE system employs a frequency hopping transceiver to combat interference and fading, but this mechanism is not employed on the advertisement transmissions. Hence, the advertising channels have been strategically chosen to reduce interference from other systems that coexist in the 2.4 GHz band. Low energy radio operation uses a Gaussian Frequency Shift Keying (GFSK) modulation to minimize transceiver complexity. The symbol rate is 1 Mbaud and supports a bitrate of 1 Mbps.

**Figure 1:**
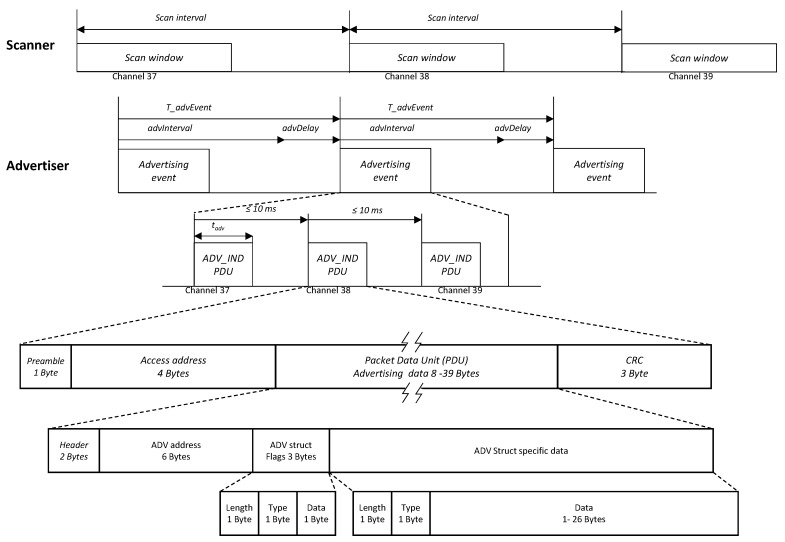
Transmissions on BLE advertising PHY channels and the frame structure.

The objective of this work is to discover and identify the devices present in the operating scenario in the shortest possible time, but with no additional data exchange. Due to this, for the purpose of the paper, only non-connectable undirected advertising events are relevant, as no connections will be established in any case. Devices that transmit advertising packets on the advertising PHY channels are referred to as advertisers, whereas the devices that enter in scanning mode, wanting to discover its neighbors without the intention of establishing a connection after the discovery, are referred to as scanners. As shown in Figure 1, in order to announce its presence, the advertiser periodically generates non-connectable undirected advertising events, which consist of a sequence of packets in the three advertising channels indexed by 37, 38 and 39. That is, the PHY channel changes for each packet sent by the advertiser in the same event following a round-robin cycle 37→38→39→37→38→39→⋯. The successive advertisement events are separated by a predefined advertisement interval. To avoid repeated collisions between simultaneous advertising devices, a random delay is added to the advertisement interval. The scanner enters into the scanning mode in periodic time intervals called the scan interval and listens to the advertising messages for a fixed time called the scan window. The scanner changes its scanning channel after each scan interval following the already explained sequence.

Depending on the type of advertising packet, the scanner may make a request to the advertiser on the same advertising PHY channel, which may be followed by a response from the advertiser. In this study, we analyze only non-connectable advertising events.

The advertising frame or advertising Packet Data Unit (PDU) follows the structure depicted in the lowest part of Figure 1. The payload is variable from eight to 39 raw bytes; however, in our experiments, the payload consists of the ADV address, a three-byte flags field to allow the reading of the advertising by standard mobile applications and the data itself. Since the transmission bitrate is 1 Mbps, Table 1 summarizes the duration of the frames for the three data sizes employed in our analysis.

### 2.1. Characterization of Non-Idealities Present in Real Devices

**Figure 2:**
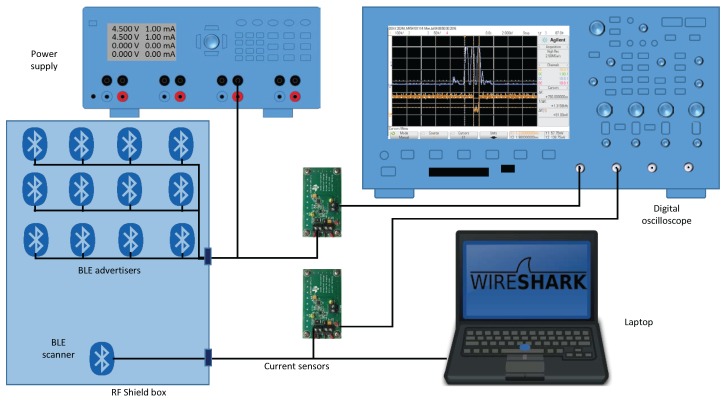
Testbed schematic setup.

From our previous experience, we know that the behavior of real devices is not ideal. For example, one could suppose that setting the same value to the scan window ($T_{scanWindow}$) and scan interval ($T_{scanInterval}$) in the scanning device would keep the receiver in a continuous scan mode. However, this is not true, and the devices present short intervals during which they are not scanning. Some of these gaps are present even when there is only the scanner in the scenario, while others appear when the scanner decodes advertising frames. These gaps should not be ignored because even if part of a frame is received during a gap, the frame would not be detected. Thus, below, we will proceed to the characterization of these gaps, differentiating the pauses exclusively due to the receiver and the ones appearing because of processing frames. To carry out this characterization, we analyzed the instantaneous power consumption of the devices using a current sensing solution based on the design of [18], which accurately detects load currents between 0 A and 1 A. Unlike the work in [15], in our case, the aim is not to analyze the power consumption of the devices, but instead, to extract behavior patterns that allow us to determine the amount of time during which a frame cannot be detected. The final implementation of the sensor testbed is shown in Figure 2.

#### 2.1.1. Scanning Gaps Caused Exclusively by the Scanning Device

We analyzed the performance of several devices of different BLE chipset manufacturers, including Broadcom, Cambridge Silicon Radio, Nordic Semiconductor, Bluegiga and Texas Instruments. We configured all of them to scan continuously, i.e., fixing the scan window and the scan interval to the same value. The reason behind this decision is to avoid other effects that would hinder the desired characterization, such as the fluctuation phenomenon described in [17,19] under specific combinations of these two values. Once the devices are characterized, analyzing the behavior for the unmatched scan window and scan interval values can be easily performed.

**Figure 3:**
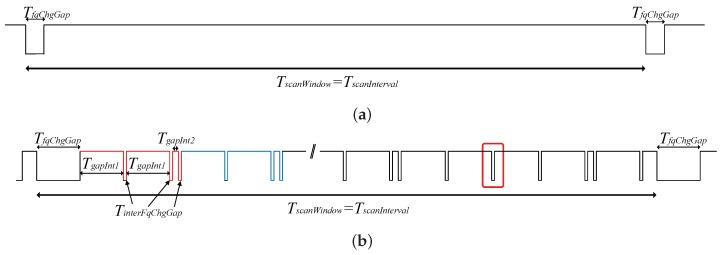
Continuous scan behavior. (**a**) Type 1 scanning devices; (**b**) Type 2 scanning devices.

From the observed measurements, we have concluded that all scanning devices follow with slight variations two behavior patterns, referred to as Type 1 and Type 2. The first pattern schematic is shown in Figure 3a, where it can be observed that a unique gap appears in every scan interval. This pause is associated with the change of the scanning frequency. It has been measured that for all Type 1 devices, this gap has a fixed duration of $T_{fqChgGap}=1.1ms$, regardless of the employed scan interval.

On the other hand, other devices follow the pattern shown in Figure 3b. As can be observed, the scanning frequency change gap is also present, but there are also other periodic pauses of less duration, which also affect the performance of the system. We will name $T_{interFqChgGap}$ the duration of these gaps and $T_{gapInt1}$ and $T_{gapInt2}$ the periods between them. It must be noted that the pattern shows a peculiar event (highlighted in red) where only one gap of duration $T_{interFqChgGap}$ is present when two of them separated by $T_{gapInt2}$ were expected. This effect happens always in the penultimate group of double gaps. The authors want to remark that the description of the device behavior presented here is based on real measurements. This behavior depends on the specific device firmware and hardware, which is designed and implemented by the chipset manufacturers.

**Figure 4:**
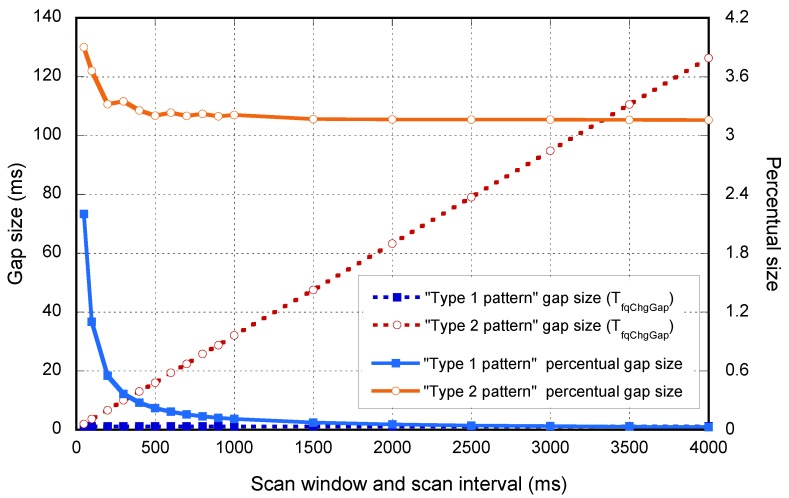
Frequency change gap duration for different scan intervals.

As an example, the numerical values for the reference device, which follows the Type 2 pattern for a $T_{scanWindow}=T_{scanInterval}=500ms$, are shown in Table 2. However, as opposed to the devices that follow the Type 1 pattern, $T_{fqChgGap}$ depends on the scan interval used, as depicted in Figure 4.

Figure 4 depicts the evolution of $T_{fqChgGap}$ versus the selected scan interval. As stated before, the $T_{fqChgGap}$ duration for the devices that follow the Type 1 pattern is constant, and the ones following the Type 2 pattern increase linearly. In the same figure, the percentage between $T_{fqChgGap}$ and the scan interval is shown, reflecting the importance of these gaps especially for low scan interval values.

#### 2.1.2. Scanning Gaps Caused by the Processing of Frames

So far, it may seem that devices following the Type 1 pattern have better performance than devices following the second pattern; however, next, we will see that global performance depends also on the number of received frames. This happens because when a frame is received, the scanner abandons momentarily the scanning state to process the frame; producing, in this way, different pauses from the already analyzed ones.

To characterize this behavior, we followed the schema depicted in Figure 2. Now, we included 14 devices manufactured by RedBearLab, specifically the BLE Nano based on Nordic nRF51822 SoC [20] as advertisers and a second current sensor for measuring simultaneously in the oscilloscope the behavior of the advertisers and the scanner. In this setup, the laptop only controlled and powered the scanner.

**Figure 5:**
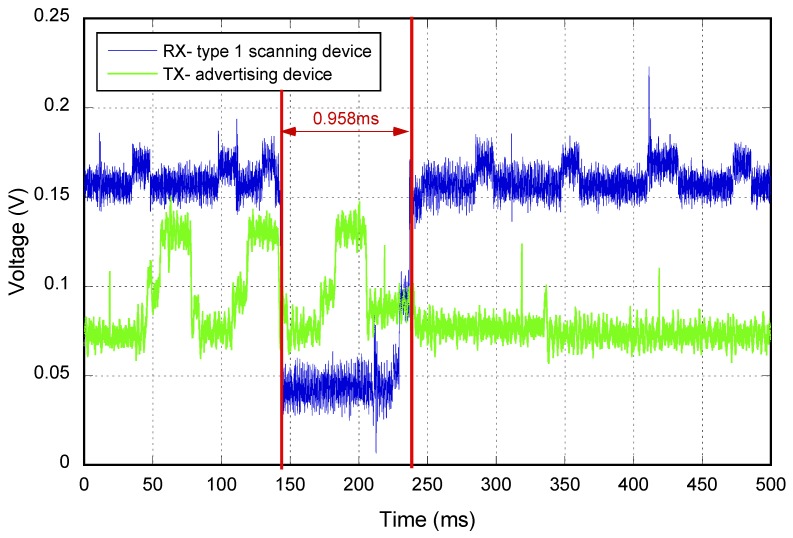
Consumption example of the advertiser/scanner.

Figure 5 is an example where the consumption of an advertiser in channels 37, 38 and 39 (green line) and the behavior of a scanner based on the Type 1 pattern (blue line) are depicted. In red, the start and the end of the gap due to the frame processing are represented. As can be observed, at that moment, the receiver was scanning Frequency 38, as it detected the second frame of the advertisement event.

These gaps are also dependent on the device. In order to determine their statistics, we implemented an automatized measurement process to analyze the duration of the pauses for different frame sizes and for the two reference devices (Type 1 and Type 2 patterns). To calculate the duration and to discard invalid samples, we apply a step detector in the transmitted and received signal. We consider the beginning of the gap when a falling edge of the transmitted signal coincides with another falling edge of the received signal within a margin of 200 samples (20 μs). The end of the gap is determined by the immediately next rising edge of the received signal. The realizations were formed by 1000 samples, and the step detector implemented was based on the Canny algorithm for one dimension [21].

**Figure 6:**
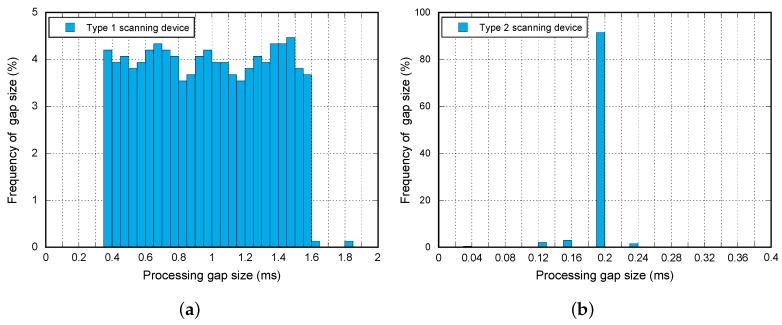
Continuous scan behavior. (**a**) Type 1 pattern; (**b**) Type 2 pattern.

From this analysis, we can extract the following conclusions depicted in Figure 6:

- The reference device with the Type 1 pattern has a uniform distributed processing gap duration ($\tau_{decodGap}$) with a minimum value ($T_{minDecodGap}$) of 350 μs and a maximum value ($T_{maxDecodGap}$) of 1.6 ms.
- For the second device (Type 2 pattern), the duration of the pause is considerably lower (194 μs) and is practically constant.
- The advertisement frame length does not have an effect in any case.

**Figure 7:**
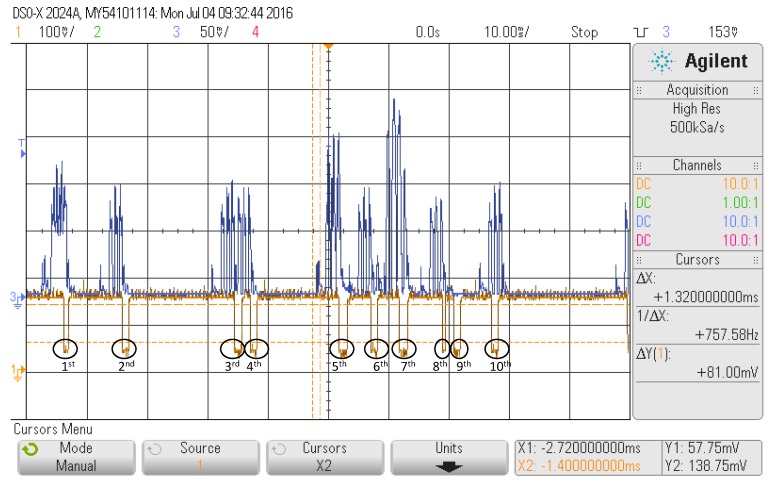
Combined effect of both types of gaps.

Figure 7 summarizes the combined effect of both gap types for the first reference device in a scenario with several advertisers (blue line). A total of ten interruptions of the scanning (yellow line) can be observed. We will analyze each of these pauses with the corresponding advertisement events prior to the gap.

All of the pauses correspond to processing gaps except from the penultimate (i.e., the ninth gap), which is due to the change of scanning frequency. At the beginning of the capture, it can be observed that the receiver is scanning at Frequency 39 because the processing frame gap is present in the last frame of the advertisement events, and after it (last gap), the detected frame is the first of the advertisement event.

If we analyze the rest of the gaps in detail, we can see that the first gap comes after an overlap of two advertisements events. From these two events, only the first is detected, while the second one is missed because the last frame of the event, which matches the current scanning frequency, arrives while the receiver is still processing the frame of the first event.

The second, third, fourth, sixth and eighth gaps correspond to the detection of single advertisements events on Frequency 39. It should be noticed that their duration is variable.

The fifth and seventh gaps also correspond to an overlap of several advertisement events, like the first. However, in the last ones, the number of advertisement events overlapping is greater attending to the amplitude depicted. In both cases, only one of the advertisements is detected, while the rest are not because the receiver is processing the detected frame.

Therefore, although the first impression is that the scanners that follow the Type 1 pattern have better performance because their scanning frequency change gaps are smaller, in scenarios with a high density of transmitters, the processing gaps gain importance, and both effects should be considered.

## 3. Analytical Model

In this section, our objective is to define a simple, but still accurate mathematical model to describe the BLE device discovering process in full accordance with the BLE specification and be able to reflect the behavior particularities of different BLE chipset manufacturers. We start from a simple long-term characterization of the collision probability according to the ideal implementation of the BLE specification. Then, we use it as a basis to derive the non-detection probability by including the required adaptations to meet the non-idealities of real BLE chipset manufacturer implementations presented in Section 2. Note that the non-detection probability depends on three main components: collisions between advertisements packets, gaps in the scanning process of the scanner BLE and Block Error Rate (BLER) due to channel and interference conditions. Next, we derive several parameters of interest, such as the mean discovery latency and mean number of detections in the time spent (dwell time) of an advertising BLE under the scanner coverage area. Finally, we prove that the proposed model agrees with experimental measurements and the results derived from simulations for a small and a high number of competing BLE devices.

The analytical model used for non-detection probability characterization takes into account the parameters and variables summarized in Table 3 and Table 4.

The number of BLE devices involved in the analysis is $N_{BLE}+1$. A fixed device is configured as a scanner, whereas the other $N_{BLE}$ devices are configured as advertisers.

In the first approach and in order to meet the requirements of applications where a number of users need to be counted in a very short period of time, the scanner BLE fixes the same scan window and scan interval values to keep the receiver scanning 100% of the time. A collision occurs when the ADV_PDU transmissions of at least two advertisers are time overlapped on the same channel that the receiver is scanning. We assume that the three channels are equally interfered and that all of the devices keep the same event advertising timings. Then, we characterize the collision probability as if the scanner were always scanning in the same channel and the advertisers were always transmitting in this frequency.

In order to develop the analytical model, we note that in both the ideal and the real implementation, the advertisement process for a device is independent of other devices, and it is not conditioned by collisions or non-detections.

At a determined time instant, the collision probability of the ADV_PDU transmission of a device with the ADV_PDU of other advertiser BLE devices is a stochastic process with memory. The advertisement event interval ($T_{advEvent}$) is the sum of a fixed component ($T_{advInterval}$) and a pseudo random variable ($\tau_{advDelay}$), and the transmission time of the ADV_PDU is fixed. Thus, the process differs from a Markovian process. The collision probability of an ADV_PDU depends on the history of transmissions of all BLE devices that coexist under the scanner coverage area. For instance, in a straightforward scenario where $N_{BLE}=2$ and the two BLE devices enter into coverage of the BLE scanner, the time difference among their first advertisements is confined to the interval $\left[0,T_{advInterval}+T_{advDelayMax}\right]$. However, if the two BLE devices have previously collided in the first transmission (1st_Adv_Tx), the time difference among their respective sequent advertisement events (2nd_Adv_Tx) is a random variable confined to the time interval $\left[0,T_{advDelayMax}\right]$. In the same way, the time difference interval between the advertisements of the two devices in a 3rd_Adv_Tx, after a collided transmission in the 1st_Adv_Tx and a non-collided transmission in the 2nd_Adv_Tx, is confined to a time interval up to $\left[0,2\;\cdot \;T_{advDelayMax}-T_{adv}\right]$, and so on.

However, in a long-term and averaged analysis, we can simplify the collision probability calculation. When a collision occurs, the probability that the transmission of a reference advertiser (started in a time instant *t*) collides with another one, $P_{noDetect}^{col,2}$, corresponds with the probability that one neighbor device starts its own transmission in the interval $\left[t-T_{adv},t+T_{adv}\right]$. As the mean time between consecutive advertisement transmissions is $T_{advInterval}+\bar{\tau_{advDelay}}$, the probability of collision is $\frac{2\;\cdot \;T_{adv}}{T_{advInterval}+\bar{\tau_{advDelay}}}$. Finally, given that transmissions of the $N_{BLE}$ devices are independent, the collision probability of a reference advertiser with any other of its neighbor devices is one minus the probability of not colliding with any of them. Thus, the collision probability in a scenario with $N_{BLE}$ advertisers is obtained with Equation (1).

(1) $$\begin{array}{c} P_{noDetect}^{col,N_{BLE}}=1-{\left(1-P_{noDetect}^{col,2}\right)}^{N_{BLE}-1}\;\mathrm{with}\;P_{noDetect}^{col,2}=\frac{2\;\cdot \;T_{adv}}{\bar{T_{advEvent}}}=\frac{2\;\cdot \;T_{adv}}{T_{advInterval}+\bar{\tau_{advDelay}}} \end{array}$$

Next, the characterization of interference from other communication systems and path loss effects can be included by means of a bler parameter on the unsuccessful reception probability of an ADV_PDU. Non-detection probability in a scenario with $N_{BLE}$ advertisers ($P_{noDetect}^{N_{BLE}}$), including collision effects and errors when ADV_PDUs do not collide, is obtained by Equation (2):

(2) $$\begin{array}{c} P_{noDetect}^{N_{BLE}}=P_{noDetect}^{col,N_{BLE}}+\left(1-P_{noDetect}^{col,N_{BLE}}\right)\;\cdot \;BLER \end{array}$$

### 3.1. Non-Detection Probability According to the Behavior of the Actual Chipsets

Now, we will include in the non-detection probability characterization the behavior particularities of different BLE chipset manufacturers. As we have seen in Section 2, the scanners present short pauses on the scanning, which we have compiled into two possible behavior patterns, shown in Figure 3. The effects of these gaps on the non-detection probability will be included in the model by treating separately the existence of the two types of pauses in which the scanner does not scan. That is:

The periodical scanning gaps (with fixed duration): which correspond to the fixed periods of time in which the scanner does not scan. They are present even when there is only the scanning device in the scenario. The non-detection probability due to these periods will be denoted by $P_{noDetect}^{scanGap}$.

The dynamic scanning gaps (with fixed or variable duration): These interruptions in the scanning process occur every time the scanner correctly detects a frame. As a consequence, they depend on the number of advertisements the scanner is detecting and thus on the number of simultaneous BLE advertisers under the scanner coverage. The non-detection probability due to these periods in a scenario with $N_{BLE}$ advertising devices will be denoted by $P_{noDetect}^{decodGap,BLE}$.

In the next sections, we describe all considerations that allow us to derive the final expression of $P_{noDetect}^{scanGap}$ and $P_{noDetect}^{decodGap,BLE}$.

#### 3.1.1. Periodical Scanning Gaps

In a first approximation, assuming that the scanner is alone, the probability of a gap of this type occurring ($P_{scanGap}^{pattern}$) is the ratio between the sum of the mean durations of all of the gaps occurring on the scan window (denoted as $\bar{T_{scanGap}}$) and the $T_{scanWindow}$. $P_{scanGap}^{pattern}$ is calculated with Equation (3), being $N_{interFqChgGap}^{scanWindow}$ the number of scattered gaps inside the $T_{scanWindow}$ and conveniently derived from using the $T_{scanWindow}$, $T_{fqChgGap}$, $T_{interFqChgGap}$, $T_{gapInt1}$ and $T_{gapInt2}$ parameters.

(3) $$\begin{array}{c} P_{scanGap}^{pattern}=\frac{\bar{T_{scanGap}}}{\bar{T_{scanWindow}}}=\frac{\bar{T_{fqChgGap}}+N_{interFqChgGap}^{scanWindow}\;\cdot \;\bar{T_{interFqChgGap}}}{\bar{T_{scanWindow}}} \end{array}$$

However, starting here, the question is what happens when simultaneous advertiser devices are present in the system and how these gaps on the scanning affect the detection. In this case, we will see that the operation mode of the scanner simplifies the characterization of this impact. We have verified that if an advertisement is sent and during the reception time, the scanner has planned a periodic gap, two possible overlapping cases can occur, with different effects:

1. If ADV_PDU transmission starts later than the start time of a periodic gap, the ADV_PDU is not detected. In this case, the time intervals between consecutive gaps ($T_{gapInt1}$ and $T_{gapInt2}$) are not affected.
2. If ADV_PDU frame reception is earlier than the start time of a periodic gap, this gap is postponed until the end of reception, regardless of whether the reception is correct, or an error, or a collision has occurred. In this case, the time interval between the postponed periodic gap and the following gap is reduced with respect $T_{gapInt1}$ or $T_{gapInt2}$, because the planned start times of the following gaps remain unchanged according to the pattern timing defined in the scanner device.

Moreover, we know that after successful advertisement receptions, the scanner introduces a gap related to the decoding time. In this case, when the reception is correct, the decoding gap and also the postponed gap scan should start simultaneously, but the scanner only applies the larger of them. On the other hand, if an unsuccessful reception or a collision occurs, the periodic scan gap is applied after the last frame involved in the collision is received.

The behavior described here is based on the analysis of real measurements of a testbed involving up to 14 simultaneous devices operating under the parameters of the standard. This behavior depends on the final manufacturer implementation of the firmware and hardware.

Furthermore, $P_{scanGap}^{pattern}$ remains unchanged, according to (3). Therefore, the non-detection probability ($P_{noDetect}^{scanGap}$), according to Equation (4), depends on the probability of generating an advertisement within the scanning gap period and is the same as Equation (3). Note that according to the characterizations made in Section 2, $T_{fqChgGap}$ and $T_{interFqChgGap}$ are usually constant.

(4) $$\begin{array}{c} P_{noDetect}^{scanGap}=\frac{\bar{T_{scanGap}}}{\bar{T_{scanWindow}}}=\frac{\bar{T_{fqChgGap}}+N_{interFqChgGap}^{scanWindow}\;\cdot \;\bar{T_{interFqChgGap}}}{\bar{T_{scanWindow}}} \end{array}$$

#### 3.1.2. Dynamic Scanning Gaps

These interruptions in the scanning process occur when the receiving device correctly detects a frame. In order to derive $P_{noDetect}^{decodGap,N_{BLE}}$, we first obtain the probability that a device causes a scanning interruption between two consecutive advertisements ($P_{decodGap}$). In the first approach, if the ADV_PDUs are always correctly detected, this corresponds to Expression (5):

(5) $$\begin{array}{c} P_{decodGap}=\frac{\bar{\tau_{decodGap}}}{T_{advInterval}+\bar{\tau_{advDelay}}} \end{array}$$

Nevertheless, since the advertisement transmission of a device has a certain probability of not being detected in the presence of $N_{BLE}$ devices (including itself), the expression to be handled in order to characterize the probability that the scanner enters in a decoding gap due to this advertiser is obtained by Equation (6):

(6) $$\begin{array}{c} P_{decodGap}=\frac{\bar{\tau_{decodGap}}}{T_{advInterval}+\bar{\tau_{advDelay}}}=\frac{\bar{\tau_{decodGap}}\;\cdot \;\left(1-P_{noDetect}^{N_{BLE}}\right)}{T_{advInterval}+\bar{\tau_{advDelay}}} \end{array}$$

Starting from $P_{decodGap}$, we obtain the probability that the scanner enters in a decoding gap in a scenario with $N_{BLE}$ advertisers. In this case, two advertisers cannot simultaneously generate a decoding gap, but in the period of time between two advertisements of a reference device, its neighbor devices may trigger $N_{BLE}-1,N_{BLE}-2,\cdots ,1$ or no decoding gaps on the scanner, depending on their detection probability. Therefore, we can calculate the mean time that the scanner generates a decoding gap within a $T_{advInterval}+\bar{\tau_{advDelay}}$ interval by multiplying the average time of the decoding gap after ADV_PDU detection ($\bar{\tau_{decodGap}}$) by the average number of devices that may be generating decoding gaps ($\bar{N_{decodGap}^{N_{BLE}}}$), according to Equation (7). Note that the system includes a finite population of advertising devices, so the probability of having *n* decoding gaps is a binomial distribution. Finally, the non-detection probability due to decoding gaps ($P_{noDetect}^{decodGap,N_{BLE}}$) is the probability of generating an advertisement within a decoding gap period, which in this case is the probability of the scanner being in a decoding gap period, given by Equation (8).

(7) $$\begin{array}{c} \bar{N_{decodGap}^{N_{BLE}}}=\sum_{n=1}^{N_{BLE}-1}n\;\cdot \;\left(\begin{array}{c} N_{BLE}-1\\ n \end{array}\right)\;\cdot \;{\left(1-P_{noDetect}^{N_{BLE}}\right)}^{n}\;\cdot \;{\left(P_{noDetect}^{N_{BLE}}\right)}^{(N_{BLE}-1)-n} \end{array}$$

(8) $$\begin{array}{c} P_{noDetect}^{decodGap,N_{BLE}}=\frac{\bar{\tau_{decodGap}}\;\cdot \;\bar{N_{decodGap}^{N_{BLE}}}}{T_{advInterval}+\bar{\tau_{advDelay}}} \end{array}$$

It is important to keep in mind that $P_{noDetect}^{decodGap,N_{BLE}}$ depends on $P_{noDetect}^{N_{BLE}}$, and at the same time, $P_{noDetect}^{decodGap,N_{BLE}}$, in addition to $P_{noDetect}^{scanGap}$, will modify the probability $P_{noDetect}^{decodGap,N_{BLE}}$. Therefore, a recursive process will be used to obtain them.

Once $P_{noDetect}^{scanGap}$ and $P_{noDetect}^{decodGap,N_{BLE}}$ are obtained, we must take into account the probability that the scanner is in a scanning gap, regardless of whether it is a decoding gap or in a gap due to its scan interruption pattern. According to the analysis made above, the two effects are characterized as independent, by they could occur simultaneously. Thus, the approach (9) is used in order to compute the non-detection probability ($P_{noDetect}^{gap,N_{BLE}}$) due to both effects.

(9) $$\begin{array}{c} P_{noDetect}^{gap,N_{BLE}}=P_{noDetect}^{scanGap}+P_{noDetect}^{decodGap,N_{BLE}}-P_{noDetect}^{scanGap}\;\cdot \;P_{noDetect}^{decodGap,N_{BLE}} \end{array}$$

Then, the probability of non-detection due to collisions and gaps $(P_{noDetect}^{gap+col,N_{BLE}}$) can be obtained with Equation (2), by setting $P_{noDetect}^{gap,N_{BLE}}$ instead of BLER.

(10) $$\begin{array}{c} P_{noDetect}^{gap+col,N_{BLE}}=P_{noDetect}^{col,N_{BLE}}+\left(1-P_{noDetect}^{col,N_{BLE}}\right)\;\cdot \;P_{noDetect}^{gap,N_{BLE}} \end{array}$$

Finally, we will use the equations above to obtain $P_{noDetect}^{N_{BLE}}$ in an iterative way according to the algorithm shown in (11).

(11) $$\begin{array}{c} n\leftarrow 0\\ P_{noDetect}^{gap+col,N_{BLE}}\left(n\right)\leftarrow P_{noDetect}^{col,N_{BLE}}\left(n\right)+\left(1-P_{noDetect}^{col,N_{BLE}}\left(n\right)\right)\;\cdot \;P_{noDetect}^{scanGap}\left(n\right)\\ n\leftarrow n+1\\ obtain\;P_{noDetect}^{decodGap,N_{BLE}}\left(n\right)\;with\;Equations\;\left(7\right)\;and\;\left(8\right)\\ P_{noDetect}^{gap,N_{BLE}}\left(n\right)\leftarrow P_{noDetect}^{scanGap}\left(n\right)+P_{noDetect}^{decodGap,N_{BLE}}\left(n\right)-P_{noDetect}^{scanGap}\left(n\right)\;\cdot \;P_{noDetect}^{decodGap,N_{BLE}}\left(n\right)\\ P_{noDetect}^{gap+col,N_{BLE}}\left(n\right)\leftarrow P_{noDetect}^{gap+col,N_{BLE}}\left(n-1\right)+\left(1-P_{noDetect}^{gap+col,N_{BLE}}\left(n-1\right)\right)\;\cdot \;P_{noDetect}^{gap,N_{BLE}}\left(n\right)\\ while\;\left(P_{noDetect}^{gap+col,N_{BLE}}\left(n\right)-P_{noDetect}^{gap+col,N_{BLE}}\left(n-1\right)\right)>\varepsilon \\ \{\\ \;n\leftarrow n+1\\ \;obtain\;P_{noDetect}^{decodGap,N_{BLE}}\left(n\right)\;with\;Equations\;\left(7\right)\;and\;\left(8\right)\\ \;P_{noDetect}^{gap,N_{BLE}}\left(n\right)\leftarrow P_{noDetect}^{scanGap}\left(n\right)+P_{noDetect}^{decodGap,N_{BLE}}\left(n\right)-P_{noDetect}^{scanGap}\left(n\right)\;\cdot \;P_{noDetect}^{decodGap,N_{BLE}}\left(n\right)\\ \;P_{noDetect}^{gap+col,N_{BLE}}\left(n\right)\leftarrow P_{noDetect}^{gap+col,N_{BLE}}\left(n-1\right)+\left(1-P_{noDetect}^{gap+col,N_{BLE}}\left(n-1\right)\right)\;\cdot \;P_{noDetect}^{gap,N_{BLE}}\left(n\right)\\ \}\\ P_{noDetect}^{gap+col,N_{BLE}}\leftarrow P_{noDetect}^{gap+col,N_{BLE}}\left(n\right) \end{array}$$

Similar to Equation (2), characterization of interference from other communication systems and path loss effects can also be included as the input bler parameter in order to compute the overall non-detection probability using (12):

(12) $$\begin{array}{c} P_{noDetect}^{N_{BLE}}=P_{noDetect}^{gap+col,N_{BLE}}+\left(1-P_{noDetect}^{gap+col,N_{BLE}}\right)\;\cdot \;BLER \end{array}$$

### 3.2. Derived Parameters of Interest

In this section, we derive several parameters of interest, such as the mean discovery latency and the mean number of detections during the time that an advertising BLE is under the scanner coverage area (dwell time), when there are $N_{BLE}$ simultaneous advertisers.

The average number of ADV_PDU transmissions required before detection ($\bar{N_{advReq}^{N_{BLE}}}$) can be straightforwardly obtained by:

(13) $$\begin{array}{c} \bar{N_{advReq}^{N_{BLE}}}=1\;\cdot \;\left(1-P_{noDetect}^{N_{BLE}}\right)+\sum_{k=1}^{\infty }\left(1+k\right)\;\cdot \;\left(1-P_{noDetect}^{N_{BLE}}\right){\left(P_{noDetect}^{N_{BLE}}\right)}^{k}\\ \;\;\;\;\;\;\;\;\;\;\;\;\;=1+\frac{P_{noDetect}^{N_{BLE}}}{\left(1-P_{noDetect}^{N_{BLE}}\right)} \end{array}$$

The average detection delay ($\bar{D_{detect}^{N_{BLE}}}$) is defined as the interval between when the advertiser transmits the first advertising packet until the scanner receives its ADV_PDU correctly. Using Equation (13):

(14) $$\begin{array}{c} \bar{D_{detect}^{N_{BLE}}}=\left(\bar{N_{advReq}^{N_{BLE}}}-1\right)\;\cdot \;\bar{T_{advEvent}} \end{array}$$

From (13), the average time between two consecutive detections ($\bar{T_{interDetect}^{N_{BLE}}}$) is given by Equation (15):

(15) $$\begin{array}{c} \bar{T_{interDetect}^{N_{BLE}}}=\bar{N_{advReq}^{N_{BLE}}}\;\cdot \;\bar{T_{advEvent}} \end{array}$$

Finally, if we define a window of opportunity (coverage time interval or dwell time), $T_{covWindow}$, the average number of detections ($\bar{N_{detect}^{N_{BLE}}}$) of an advertiser BLE will be:

(16) $$\begin{array}{c} \bar{N_{detect}^{N_{BLE}}}=\frac{T_{covWindow}}{\bar{T_{interDetect}^{N_{BLE}}}} \end{array}$$

## 4. Experimental and Simulation Validation of the Analytical Model

The device discovery process for BLE as described in Section 2 is fairly simple, and in fact, this is the reason why we want to explore potential benefits of using this process in applications where a certain number of users need to be counted in a very short period of time, as the sport applications referred to in Section 1. According to the requirements of these application scenarios, we want to quantify the discovery process for a large number of devices. The main parameters of the performance evaluation include:

- The collision probability/non-detection probability.
- The average discovery latency or, alternatively, the mean time between consecutive ADV_PDU detections of an advertiser.
- The mean number of ADV_PDU detections within a window of opportunity time (coverage time interval or dwell time).

Additional parameters, such as the probability that all coexisting advertisers can be detected by the scanner within the coverage time, are also included. Even in ideal conditions, with respect to wireless channel effects or interference from other systems, the performance of the device discovery process is greatly influenced by the scanner parameter settings, such as the scanning interval, the scan window size and, in the advertisers, the advertising interval and the advertising PDU size. Specifically, concerning the scanning device, by choosing the scanning interval and the scan window size, we can control the tradeoff between the discovery capability and the energy consumption of the scanner. That is, for a given scanning interval, shortening the scan window, the energy consumption of the scanner is decreased, whereas the non-detection probability of the advertising devices increases. Nevertheless, from the point of view of these specific applications, the energy consumption is not one of the most relevant parameters, given that the main objective is to detect a high number of BLE devices in a short time window, even if we detect the identifier of the same device several times. Repetition will provide us a desirable redundancy in order to compensate even high BLER scenarios that extend the experimental conditions and simulation analysis performed here. Thus, as explained in Section 2.1.1, to avoid other effects that would make the desired characterization difficult, we fixed $T_{scanInterval}\;=\;T_{scanWindow}$. Note that wireless data transmission is affected by many physical and environmental conditions, which degrade the behavior of the system in real-life conditions. This is the reason why the experiments, simulations and analysis are performed in a controlled scenario without interferences and with the best channel conditions. Once we verify that the numerical results from the mathematical model closely match those obtained in the simulation and testbed measurements, extensive experiments could be performed with BLE devices in several channel/interference conditions, which could be easily included in the mathematical model through the appropriate BLER values.

Related to the advertisers, several previous works [17] focus on the performance impact in terms of the tradeoff between consumption and discovery latency, connected with the relationship between $T_{advInterval}$ and $T_{scanWindow}$, within a scenario where $T_{scanWindow}<T_{scanInterval}$. However, in our case, given that continuous scanning is assumed, the main objective is to verify how the real peculiarities of scanning patterns of the BLE chipsets affect the discovery process and, even, notably modify the ideal expected performance. Regarding the $T_{advInterval}$ duration influence, it is evident that larger $T_{advInterval}$ values result in lower energy consumption on the advertiser side and lower collision probability at the scanner. Nevertheless, the mean number of possible detections on a short window of opportunity decreases or even becomes zero. As we said above, in some scenarios, redundancy by repetition could be beneficial. Thus, we want to quantify for an increased number of simultaneous advertisers the impact of $T_{advInterval}$ in the non-detection probability, the discovery delay and the mean number of detections. About ADV_PDUs sizes, it seems also clear that shorter ADV_PDUs will result in lower collision probabilities, whereas longer ADV_PDUs allow one to extend the capabilities of the system; for instance, monitoring physiological parameters in a sporting event (for example, averaged health parameters, such as temperature, pulse/heart rate, oxygen saturation), that only require a few bytes in order to record global statistics or setup health alarms concerning a specific participant. Thus, it is also interesting to quantify the discovery performance connected with the ADV_PDU size for several $T_{advInterval}$ settings.

Summarizing, the objectives of the evaluations performed in this section are:

- To validate the characterization performed in Section 2 of the non-idealities present on the scanning process by comparing simulations and experiment tests with an increasing number of BLE advertisers and different parameter settings.
- To analyze how close the numerical results from the mathematical model match those obtained in the simulations and experimental tests for any parameter values specified in the standard and also considering the real chipset behavior. This ratifies the potential benefits of using a simple analytical model in order to easily obtain an upper bound of discovery capacities in a potential application scenario.
- To carry out an analysis in ideal channel conditions that allows one to evaluate the non-detection probability, delay, etc., as the number of BLE devices increases for several parameter settings and real chipsets.

### 4.1. Testbed Conditions

**Figure 8:**
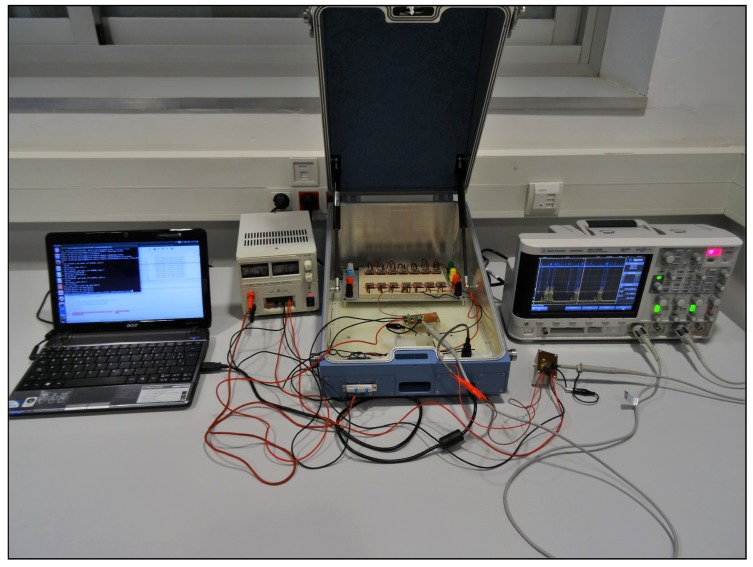
Experimental testbed.

We performed a series of tests in the scenario following the schema depicted in Figure 2. All of the devices, up to 14 advertisers and one scanner, were placed inside an RF-shield box, always with the same arrangement; see Figure 8. The scanner was connected to a laptop where we performed the raw capture of the data using Tshark.

Tshark is the command line tool of Wireshark [22], a common network protocol analyzer usually employed to audit local area networks, but which can also be used to analyze frames captured through a Bluetooth interface. We then processed the raw data, filtering by interface and advertiser in order to extract different statistics and calculate the non-detection probability and time between consecutive detections. With this configuration, the scanners were programmed with a 500-ms value for both the scan window and scan interval. The advertisers had a wider variation of configurations. The advertisement interval, $T_{advInterval}$, and the size of the advertisement data are set according to the evaluation conditions considered in Section 4.3. Once the number of selected devices were programmed, we double checked their configuration with the android application of Nordic Semiconductor, nRF Connect for Mobile [23]. Finally, we closed the RF-shield box and proceeded with the measurements.

The transmission power of advertising devices could be configured in a range between −40 dBm and 4 dBm. For this range of values, we checked that there was no impact on the BLER in our scenario because the propagation losses are very low inside the RF-shield box. In order to observe some variation on the BLER, it was necessary to insert an extra attenuation shielding the advertising device. The final employed value for all devices was 4 dBm. We captured data during three hours for each parameter combination set.

### 4.2. Simulation

In order to validate both the analytical model and experimental results, we developed a BLE simulation framework in C++ that fully complies with the BLE advertisement specification. We also included two scanner configurations with the scan patterns identified in Section 2. The simulations were conducted without any of the simplifications or assumptions used to derive the analytical model. In order to obtain the desired statistics, the discovery process was monitored in a number of coverage time intervals. In the simulation, the initial instant for each coverage time interval ($T_{covWindow}$) was randomly chosen at any continuous time between $\left[0,T_{scanWindow}\right]$, and in the same way, the starting transmission time of each advertising device was independently generated between $\left[0,T_{advInterval}+T_{advDelayMax}\right]$. A number up to 50,000 coverage time intervals was considered when $N_{BLE}$ is lower than 20 and up to 10,000 for a higher number of devices. Simulation allows including errors due to channel or interference conditions. However, the results are obtained without considering any reception failure of ADV_PDUs (i.e., BLER = 0%) in order to meet experimental testbed conditions.

### 4.3. Performance Results

**Figure 9:**
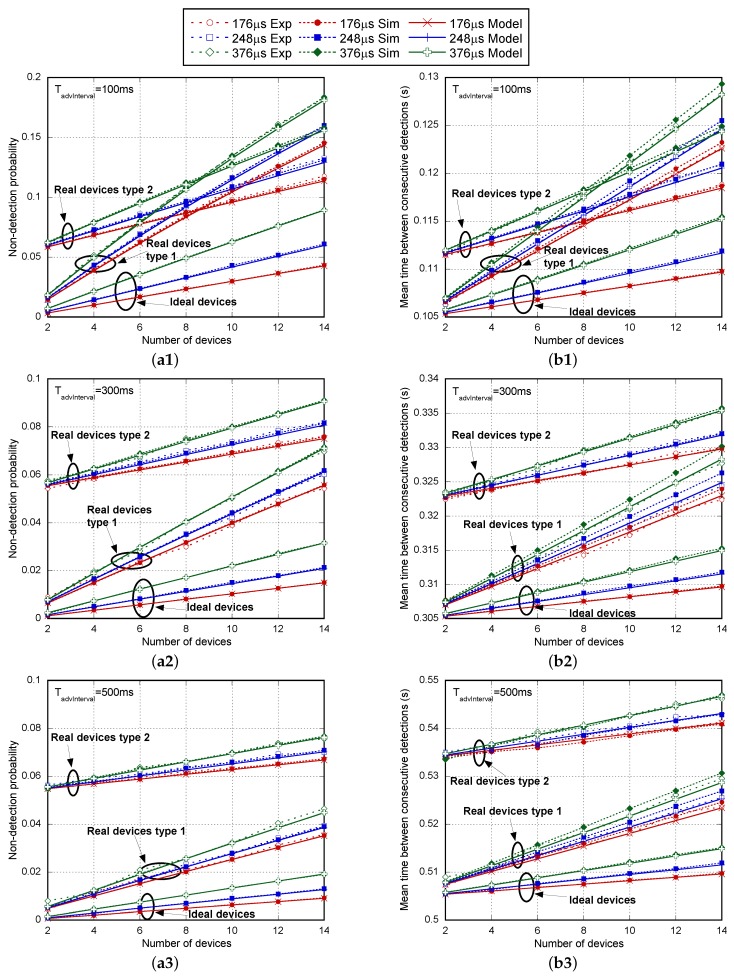
Non-detection probability (**a1**–**a3**) and mean time between consecutive detections in seconds (**b1**–**b3**) as the number of BLE advertisers increases, for several $T_{adv}$ values (176 μs, 248 μs and 376 μs) and for different $T_{advInterval}$ intervals (100 ms, 300 ms and 500 ms) with $T_{advDelayMax}=10ms$. Comparison among experimental measurements, simulation and the analytical model.

The evaluation has been conducted using the setting as listed in Table 5. Figure 9 compares the analytical model (Model) and the simulation results (Sim) obtained for the ideal implementation of the standard to those obtained when real scanning devices are assumed, including in the last case, the results obtained in the experimental test (Exp) for a number of $N_{BLE}$ devices up to 14. The transmission power of advertising devices was set to 4 dBm, and it has been verified that in this case and even when power is set to −40 dBm, the non-detection probability for only one advertising device corresponds to $P_{noDetect}^{scanGap}$. Therefore, BLER effects are almost negligible and enable the comparison of results between the model and simulations in ideal channel conditions. Figure 9a1–a3 shows the non-detection probability for all of these cases, as the number of advertiser BLEs increases from two to 14 for several $T_{adv}$ values (176 μs, 248 μs and 376 μs) and for different $T_{advInterval}$ intervals (100 ms in Figure 9a1, 300 ms in Figure 9a2 and 500 ms in Figure 9a3 with $T_{advDelayMax}=10ms$). From these figures, it is observed that the numerical results from the mathematical model match with the simulation ones for both ideal and real implementations and for any parameter setting. At the same time, it seems clear that model and simulations perfectly match the experimental results, thus validating the characterization of the scanner process in the actual chipsets.

We can see how non-detection probability increases with the number of simultaneous advertising devices under the scanner coverage and also with higher $T_{adv}$ and $T_{advInterval}$ values. By comparing the results, we can observe that differences between the ideal standard and the actual implementations are quite significant. It is evident that the assumption of the ideal case implies a clear underestimation of non-detection probability. On the other hand, differences between chipsets from different manufacturers need to be taken into account. In this particular case, the Type 1 scanning device provides better results than the Type 2 up to $N_{BLE}=8$ when $T_{advInterval}$ is equal to 100 ms. This advantage is maintained up to $N_{BLE}=21$ when $T_{advInterval}$ is equal to 300 ms for $T_{adv}=376\mu s$ or up to 23 for $T_{adv}=176\mu s$. Finally, the Type 1 device is superior up to $N_{BLE}=35$ when $T_{advInterval}$ is equal to 500 ms with $T_{adv}=376\mu s$ and up to 37 for $T_{adv}=176\mu s$.

**Figure 10:**
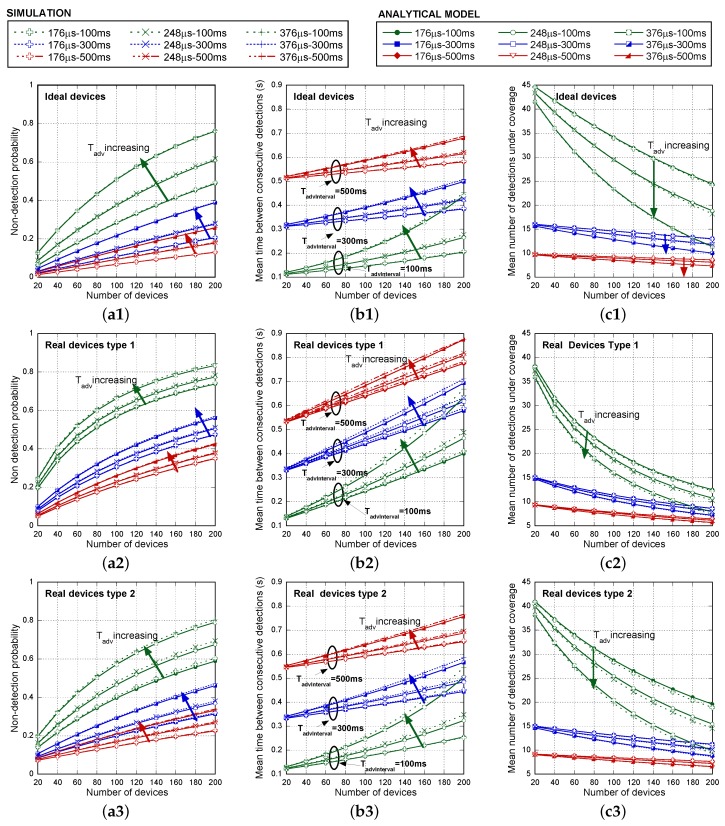
Non-detection probability (**a1**–**a3**), mean time between consecutive detections in seconds (**b1**–**b3**) and mean number of detections under coverage ($T_{covWindow}=5$ s) (**c1**–**c3**), as the number of advertisers increase, for several $T_{adv}$ values (176 μs, 248 μs and 376 μs) and for different $T_{advInterval}$ intervals (100 ms, 300 ms and 500 ms) with $T_{advDelayMax}=10ms$. Comparison between simulation and the analytical model for ideal (a1,b1,c1), Type 1 real devices (a2,b2,c2) and Type 2 real devices (a3,b3,c3).

However, as is shown in Figure 10, the Type 2 scanning device is a better choice when a high number of advertising devices coexist. In similar conditions, the mean time between consecutive detections is shown in Figure 9b1–b3. This parameter has been chosen instead of the average detection delay because this statistic can be directly obtained by processing the data from experimental measurements without any kind of assumption. We can compare approximately the average detection delay only by subtracting $\bar{T_{advEvent}}$ from the mean time between advertisements. As shown in the figures, results from the mathematical model, simulation and experimental tests match again for both ideal and real implementations for any parameter setting. Focusing on the ideal standard implementation, it is easy to check that, as expected, the average detection delay is clearly higher as long as $T_{advInterval}$ decreases due to the higher non-detection probability. Comparing results for $N_{BLE}=14$ and $T_{adv}=376\mu s$, they are almost similar. For instance, for ideal devices, the average detection delay is 10.28 ms when $T_{advInterval}=100ms$ (in this case, $\bar{T_{advEvent}}=105ms$), 9.94 ms when $T_{advInterval}=300ms$ ($\bar{T_{advEvent}}=305ms$) and 9.87 ms when $T_{advInterval}=500ms$ ( $\bar{T_{advEvent}}=505ms$). The absolute values of non-detection probability are not high enough to have a notable impact on this parameter. However, differences in the mean time between consecutive detections will have a great impact in the number of potential detections in a fixed coverage time interval ($T_{covWindow}$), being a valuable parameter for the intended applications.

Figure 10 extends the comparison for a larger number of advertising devices, up to 200. In this case, only simulation and mathematical results are illustrated related to the non-detection probability (Figure 10a1–a3), the mean time between consecutive detections (Figure 10b1–b3) and the mean number of detections under coverage (Figure 10c1–c3), as the number of advertisers increases, for several $T_{adv}$ values (176 μs, 248 μs and 376 μs) and for different $T_{advInterval}$ with $T_{advDelayMax}=10ms$. The mean number of detections under coverage evaluation only intends to be illustrative. Discovery capacity needs to be evaluated for each potential application, being $T_{covWindow}$ particularized to the expected value. In this case, as an example, $T_{covWindow}$ is set to 5 s.

**Figure 11:**
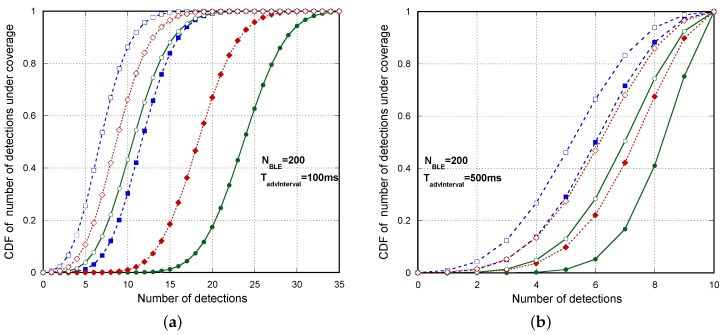
cdf of the number of detections under coverage ($T_{covWindow}=5s$) for several $T_{adv}$ values ($T_{adv}=176\mu s$, $T_{adv}=376\mu s$) when $T_{advInterval}=100ms$ (**a**); and $T_{advInterval}=500ms$ (**b**) with $T_{advDelayMax}=10ms$ and when there are $N_{BLE}=200$ BLE advertisers. Comparison between simulation results for ideal devices, Type 1 real devices and Type 2 real devices.

In order to facilitate the visualization of results, unlike Figure 9, Figure 10 compares separately simulation and the analytical model for ideal, Type 1 scanning devices and Type 2 scanning devices. We show that mathematical curves almost coincide with the simulation results for the entire range of parameters and devices. Differences between actual devices are significant. As anticipated before, for a larger number of advertising devices, Type 2 scanning devices clearly offer better results. Note that, when a few advertisers are considered, the impact of the additional micro-gap scans exceeds that caused by gaps due to decoding. However, as long as the number of advertisers increases, decoding interruptions are the most challenging issue in the successful detection probability. In this case, mean $\tau_{decodGap}$ is clearly higher in Type 1 devices. Concerning the averaged detection delay, in contrast to Figure 9, in Figure 10b1–b3, we clearly check (see $N_{BLE}=200$) that the averaged detection delay is higher as long as decreases, as expected. However, shorter $\bar{T_{advEvent}}$ remains to be desirable to achieve a lower mean time between consecutive detections and, thus, a higher number of detections in a $T_{covWindow}$. Concerning this last parameter (see Figure 10c1–c3), we can see that using this simple discovery process, the number of potential detections is high enough even for a large number of simultaneous devices, providing a high margin for repetitions in order to combat real propagation and interference conditions with non-negligible BLER. Figure 11a shows the Cumulative Density Function (CDF) of the number of detections for several $T_{adv}=176\mu s$, $T_{adv}=376\mu s$ with $T_{advInterval}=100ms$ and $T_{advDelayMax}=10ms$, when there are $N_{BLE}=200$ BLE advertisers. We can see that, even in the worst case (real device Type 1 and $T_{adv}=376\mu s$), less than 7% of the advertisers are detected three or less times. In this same case, the probability of zero detections of a device is 0.000692. Using this probability, we can theoretically derive in a simple way the probability that not all of the devices (200) are detected in $T_{covWindow}$, using the expression $1-{\left(1-0.000692\right)}^{200}$. This probability is around 13%, which agrees with the result obtained by the simulation, as shown in Table 6. Nevertheless, in most of the cases, non-detection affects only one or two devices, as the non-detection probability of a BLE in $T_{covWindow}$ is very low. In a similar way, Figure 11b shows the cdf of the number of detections for $T_{adv}=176\mu s$, $T_{adv}=376\mu s$ with $T_{advInterval}=500ms$ and $T_{advDelayMax}=10ms$, when there are $N_{BLE}=200$ BLE advertisers. Now, in the worst case (Type 1 real device and $T_{adv}=376\mu s$), less than 13.4% of the advertisers are detected three or less times. Table 6 shows the simulation results concerning the probability that not all of the devices (200) are detected in the $T_{covWindow}$ for $T_{adv}=176\mu s$, $T_{adv}=376\mu s$ with $T_{advInterval}=100ms$ and $T_{advInterval}=500ms$ when $T_{advDelayMax}=10ms$.

## 5. Conclusions

In this paper, we have experimentally studied the BLE discovery process when a high number of users needs to be discovered in a short period of time. After analyzing several scanning devices from different BLE chipset manufacturers, we have shown that all of them have non-idealities in the scanning process. Even though this aspect is not taken into account in other studies, we have demonstrated that these non-idealities have a severe impact on the performance of the discovery process. We have identified two main behavior patterns that characterize the diverse actual devices by setting appropriate parameter values. Once these non-idealities have been characterized, an extensive performance analysis for a varying number of devices and feasible parameter combinations concerning the advertising interval and advertising frame size has been conducted. Simulations and experimental measurements for two specific chipsets have been compared, considering up 14 devices. This study has been extended by simulation for a higher number of devices. It has been observed that the advantages of using a particular scanner chipset over another depend on the number of devices that need to be detected, and they are also conditioned by the selected parameter settings.

This work also includes a simple model that meets both the standard implementation and the described peculiarities of the actual implementation on the chipsets. The results from the mathematical model, the simulation results and the experimental measurements of real devices closely match for any feasible parameter values specified in the standard and for any number of simultaneous advertising devices under a scanner coverage. That is, the model can be useful in order to easily obtain an upper bound for the discovery capacity and to select the desirable parameters values according to a particular BLE application.

## Figures and Tables

**Table 1:** Frame duration for the employed sizes.

Data Bytes	Total Frame Bytes	Duration
1	22	176 ms
10	31	248 ms
26	47	376 ms

**Table 2:** Parameter values for a continuous scan with $T_{scanWindow}=T_{scanInterval}=500ms$.

Parameter	Value	
	Type 1 Pattern	Type 2 Pattern
$T_{fqChgGap}$	1.1 ms	16.05 ms
$T_{interFqChgGap}$	-	300 μs
$T_{gapInt1}$	-	16.82 ms
$T_{gapInt2}$	-	4.3 ms

**Table 3:** Parameters included in the model.

Parameter	Description
$T_{adv}$	Transmission time of the advertisement PDU (ADV_PDU)
$T_{advInterval}$	Fixed advertisement interval
$T_{advDelayMax}$	Maximum value of the random backoff (standard: 10 ms)
$\tau_{advDelay}$	Random backoff between advertisements: $Uniform[0,T_{advDelayMax}]$
$T_{advEvent}$	Advertisement event interval: $T_{advInterval}+\tau_{advDelay}$
$T_{scanInterval}$	Scan interval
$T_{scanWindow}$	Scan window
$T_{fqChgGap}$	Gap due to change of scanning frequency (Type 1 and 2 scanners)
$T_{interFqChgGap}$	Duration of scattered gaps inside the scan interval (Type 2 scanner)
$T_{gapInt1},T_{gapInt2}$	Time intervals between scattered gaps inside the scan interval (Type 2 scanner)
$T_{minDecodGap}$	Minimum value of processing gap after ADV_PDU detection
$T_{maxDecodGap}$	Maximum value of processing gap after ADV_PDU detection
$\tau_{decodGap}$	Processing gap after ADV_PDU detection. $Uniform[T_{minDecodGap},T_{maxDecodGap}]$
$T_{scanGap}$	Sum of durations of all the gaps occurred in the scan window
$N_{interFqChgGap}^{scanWindow}$	Number of scattered gaps inside the $T_{scanWindow}$
$N_{BLE}$	Total number of advertising devices that are in the coverage area of the scanner
	and can be potentially colliding

**Table 4:** Variables included in the mathematical model.

Variable	Description
$P_{scanGap}^{pattern}$	Probability that a periodical scanning gap occurs
$P_{decodGap}$	Probability that a device causes a scanning interruption
	between two consecutive advertisements
$\bar{N_{decodGap}^{N_{BLE}}}$	Average number of devices that may be generating decoding gaps
	within a $T_{advInterval}+\bar{\tau_{advDelay}}$ interval in a scenario with $N_{BLE}$ advertisers
$P_{noDetect}^{scanGap}$	Non-detection probability due to periodical scanning gaps
$P_{noDetect}^{decodGap,N_{BLE}}$	Non-detection probability due to dynamic scanning gaps (decoding gaps)
	in an scenario with $N_{BLE}$ advertisers
$P_{noDetect}^{gap,N_{BLE}}$	Non-detection probability due to scanning gaps
	(periodical and dynamic scanning gaps) in a scenario with $N_{BLE}$ advertisers
$P_{noDetect}^{col,N_{BLE}}$	Non-detection probability of a device due to collisions
	in a scenario with $N_{BLE}$ advertisers
$P_{noDetect}^{gap+col,N_{BLE}}$	Non-detection probability of a device due to collisions and gaps
	in a scenario with $N_{BLE}$ advertisers
$P_{noDetect}^{N_{BLE}}$	Overall non-detection probability of a device due to collisions,
	gaps and channel errors in a scenario with $N_{BLE}$ advertisers
$\bar{N_{advReq}^{N_{BLE}}}$	Average number of ADV_PDU transmissions required before detection
	of a device in a scenario with $N_{BLE}$ advertisers
$\bar{D_{detect}^{N_{BLE}}}$	Average detection delay of a device in a scenario with $N_{BLE}$ advertisers
$\bar{t_{interDetect}^{N_{BLE}}}$	Average time between two consecutive detections of a device
	in a scenario with $N_{BLE}$ advertisers
$\bar{N_{detect}^{N_{BLE}}}$	Average number of detections of an advertiser BLE within a window of
	opportunity (coverage time interval or dwell time) in a scenario with $N_{BLE}$ advertisers

**Table 5:** Parameters used in the evaluation.

General Parameters		Real Scanner Service Parameters		
Parameter	Values	Parameter	Value	
			Type 1 Pattern	Type 2 Pattern
$T_{adv}$	176 μs, 248 μs, 376 μs	$T_{fqChgGap}$	1.1 ms	16.05 ms
$T_{advInterval}$	100 ms, 300 ms, 500 ms	$T_{interFqChgGap}$	-	300 μs
$\tau_{advDelay}$	$Uniform(0,T_{advDelayMax})$	$T_{gapInt1}$	-	16.82 ms
$T_{advDelayMax}$	10 ms	$T_{gapInt2}$	-	4.3 ms
$T_{scanInterval}$	500 ms	$\tau_{decodGap}$	$Uniform(T_{minDecodGap},T_{maxDecodGap})$	
$T_{scanWindow}$	500 ms	$T_{minDecodGap}$	350 μs	194 μs
$N_{BLE}$	2 to 200	$T_{maxDecodGap}$	1.6 ms	194 μs
$T_{covWindow}$	5 s			

**Table 6:** Probability (in %) that not all of the devices (200) are detected in the $T_{covWindow}$ (5 s) for several $T_{adv}$ values ($T_{adv}=176\mu s$, $T_{adv}=376\mu s$) when $T_{advInterval}=100ms$ and $T_{advInterval}=500ms$ with $T_{advDelayMax}=10ms$ and when there are $N_{BLE}=200$ BLE advertisers. Comparison between simulation results for ideal devices, Type 1 real devices and Type 2 real devices.

$T_{advInterval}$	$T_{\mathrm{adv}}=176\;\mu s$			$T_{\mathrm{adv}}=376\;\mu s$		
	Ideal	Type 1	Type 2	Ideal	Type 1	Type 2
100 ms	0 %	0.04 %	0 %	0.2 %	12.96 %	2.52 %
500 ms	0 %	4.94 %	0.54 %	0.3 %	19.69 %	6.48 %

## Abbreviations

The following abbreviations are used in this manuscript:

ADV	Advertising
BLE	Bluetooth Low Energy
BLER	Block Error Rate
CDF	Cumulative Density Function
GFSK	Gaussian Frequency Shift Keying
ISM	Industrial Scientific Medical
IoT	Internet of Things
LTE-M	LTE Machine to Machine
NB-IoT	Narrowband IoT
PDU	Packet Data Unit
PHY	Physical
SoC	System on a Chip
